# A bibliometric analysis of the research hotspots and frontiers related to cell death in spinal cord injury

**DOI:** 10.3389/fneur.2023.1280908

**Published:** 2024-01-05

**Authors:** Kelin He, Han Yu, Jieqi Zhang, Lei Wu, Dexiong Han, Ruijie Ma

**Affiliations:** ^1^Department of Acupuncture and Moxibustion, The Third Affiliated Hospital of Zhejiang Chinese Medical University (Zhongshan Hospital of Zhejiang Province), Hangzhou, Zhejiang, China; ^2^Key Laboratory of Acupuncture and Neurology of Zhejiang Province, The Third School of Clinical Medicine (School of Rehabilitation Medicine), Zhejiang Chinese Medical University, Hangzhou, Zhejiang, China

**Keywords:** spinal cord injury, cell death, apoptosis, bibliometric analysis, research hotspots

## Abstract

**Background:**

Spinal cord injury (SCI) is a severe central nervous trauma that can cause serious consequences. Cell death is emerging as a common pathogenesis after SCI. In the last two decades, numerous studies have been published in the field of cell death after SCI. However, it is still rare to find relevant bibliometric analyses. This bibliometric study aims to visually represent global research trends in the field of cell death after SCI.

**Methods:**

Bibliometric data were sourced from the Web of Science Core Collection (WoSCC) database. VOSviewer, CiteSpace, and R software (“bibliometrix” package) were used to analyze and visualize bibliometric data. Annual scientific production, countries/regions, institutions, authors, journals, highly cited papers, keywords, and literature co-citation were evaluated to determine research performance.

**Results:**

An analysis of 5,078 publications extracted from the WoSCC database revealed a fluctuating yet persistent growth in the field of cell death after SCI over the past 23 years. China and the United States, contributing 69% of the total publications, were the main driving force in this field. The Wenzhou Medical University from China contributed to the most papers. In terms of authors, Salvatore Cuzzocrea from the University of Messina had the highest number of publications. The “Journal of Neurotrauma” was the top journal in terms of the number of publications, however, the “Journal of Neuroscience” was the top journal in terms of the number of citations. The theme of the highly cited articles mainly focused on the mechanism of cell death after SCI. The keyword and literature co-citation analysis mainly focused on the mode of cell death, mechanism research of cell death, and functional recovery after SCI.

**Conclusion:**

This study analyzes the research hotspots, frontiers, and development trends in the field of cell death after SCI, which is important for future studies.

## 1 Introduction

Spinal cord injury (SCI) is a trauma to the central nervous system that can cause serious consequences. SCI often occurs in traffic accidents, falls, or violence (1), and often leads to a partial or complete disruption of brain–body communication below the level of injury. As a result of disruption of the descending motor tracts, paralysis is one of the most common symptoms of SCI. In addition, SCI can also cause pain, autonomic dysreflexia, spasticity, and a loss of respiratory, bladder, bowel, and sexual functions. Consequently, SCI patients have a severely reduced quality of life. SCI is characterized by the delayed onset of secondary lesions, with cell death affecting the initially spared neighboring tissue through complex mechanisms (2–4). Cell death, particularly affecting neurons, glial (5), is always the outcome of SCI. After a primary SCI, cell death may have a critical effect on secondary injury mechanisms that cause the ultimate neurological deficits. Hence, understanding the molecular basis of SCI may be beneficial for improved neuronal, and glial survival, and reduce neurological deficits.

Cell death plays a pivotal role in the regulation of developmental processes, maintenance of homeostasis, and orchestration of immune responses in multicellular organisms. Moreover, its dysregulation has been implicated in a range of pathological conditions. Cell death can be categorized into two distinct types (6): accidental cell death, which occurs rapidly and lacks discernible involvement of specific molecular machinery such as necrosis, and regulated cell death, which is governed by intracellular molecular mechanisms such as apoptosis and can be modulated through pharmacological and genetic interventions (7). Apoptosis has been widely acknowledged for its role in eliminating dysfunctional cells under both physiological and pathological circumstances. Studies have demonstrated that attenuating apoptosis in spinal cord neurons can enhance neurological function following SCI (8, 9). Furthermore, SCI encompasses a range of regulated cell death mechanisms, such as necroptosis, autophagy, paraptosis, pyroptosis, and ferroptosis (10). Necroptosis, a recently discovered form of programmed necrosis, arises from various extracellular and intracellular stimuli. Multiple experimental investigations have substantiated the notion that the inhibition of necroptosis can yield protective outcomes in the context of SCI (11, 12). Furthermore, autophagy assumes a pivotal role in upholding a state of healthy and stable homeostasis in SCI. Prior research indicates that autophagy can be genetically or pharmacologically manipulated, thereby playing a significant role in neurological recovery (13). Moreover, ferroptosis, pyroptosis, and paraptosis emerge as crucial contributors to the promotion of neurological function recovery following SCI (10). Due to its crucial role in the progression of diseases, cell death emerges as a prospective therapeutic target in the context of SCI.

Bibliometrics quantitatively and qualitatively assesses research and scientific progress (14, 15). It identifies and visualizes, established fields of study through literature analysis (16–18). Regarding a particular research area, bibliometrics employs mathematical and statistical tools to map hot topics and trends. Over the past decades, research in the field of cell death after SCI has developed rapidly, with several high-quality papers published (10, 11, 19). Active bibliometric research in this area may facilitate further progress. Currently, a bibliometric analysis of research in the field of cell death after SCI has not been conducted. Hence, the purpose of this study is to use bibliometric analysis to analyze the trends and hot topics in the field of cell death after SCI. Research interests will be identified, and future research directions will be influenced by this systematic mapping of research progress regarding the field of cell death after SCI.

## 2 Methods

### 2.1 Sources of data and search strategies

In light of the literature’s quality and the necessity for adhering to proper referencing standards, we opted to utilize the Science Citation Index-Expanded from the Web of Science Core Collection (WoSCC) as our primary data source. The WoSCC, which encompasses over 12,000 high-quality scientific journals, is renowned for hosting a relatively reliable database. It has also been regarded as the preferred database in previous bibliometric studies (20–23). To generate the initial search result, the following query was used under the reference of MeSH and published literatures (10, 24): ([Topic = Ferroptosis] OR [Topic = Parthanatos] OR [Topic = Paraptosis] OR [Topic = Pyroptosis] OR [Topic = Necroptosis] OR [Topic = Mitochondrial Transmembrane Permeability-Driven Necrosis] OR [Topic = Necrosis] OR [Topic = Immunogenic Cell Death] OR [Topic = Autophagic Cell Death] OR [Topic = Autophagy] OR [Topic = Apoptosis] OR [Topic = Cell Death]) AND ([Topic = Spinal Cord Injury] OR [Topic = Spinal Cord Injuries]); Our period of interest was from 1997 to 2022. To mitigate the substantial bias resulting from frequent database updates, two investigators (YH and ZJQ) independently conducted all searches; both queries were performed on 3 August 2023.

### 2.2 Data extraction and collection

The data for this study was obtained from WoSCC through the utilization of the “Export Records to File” feature, wherein the record content was configured to include both the full record and cited references. The resulting data was exported in plain text to enable subsequent analysis. The bibliographic details, encompassing information such as the annual publication, countries, institutions, authors, journals, high cited articles, keywords, and references, were then condensed and stored in a Microsoft Excel file (Version: 2021). This study utilized VOSviewer (Version 1.6.20), CiteSpace (Version 6.2.R4), and R software (Version 4.3.2) along with the R packages “bibliometrix” to perform both qualitative and quantitative analyses. The Journal Citation Reports (JCR) 2022 provided the impact factor (IF) and quartile for a journal category. In cases of disagreement, resolution was sought through the assessment of a third senior reviewer (MRJ).

### 2.3 Bibliometric analysis

The software tool VOSviewer, which was developed by Van Eck and Waltman from Leiden University in 2009, has been widely utilized for the visualization and construction of bibliometric network maps (25). In this study, VOSviewer was employed to visually depict the co-authorship analysis of countries, institutions, authors, and co-citation analysis of journals, co-occurrence analysis of keywords, and literature co-citation analysis. The study employed the “fractional counting” method for counting, and multiple VOSviewer thesaurus files were utilized to merge different variants. In addition，the VOSviewer was instrumental in generating three distinct visualization maps, namely the network, density, and overlay visualization maps, each conveying unique implications. In these comprehensive knowledge maps, nodes represent elements such as countries, institutes, or authors, while the links between nodes depict the relationships between these elements. Distinct colors were assigned to the nodes and lines based on various clusters or the average appearing year (26).

The bibliometric tool CiteSpace, developed by Chen Chaomei from Drexel University, was utilized in this study to examine the dynamics of progression and research clusters within specific subjects (27). CiteSpace was employed to visually analyze the keyword clusters and keywords with the highest citation bursts. The parameters used in CiteSpace were as follows: the period was set from 1 January 2000 to 31 December 2022; the year of slice was set to 1; the selection criteria for the g-index was set at k = 25; the selection criteria for the top 20 was used; the link retaining factor was set at LRF = 3; the look back years were set at LBY = 5; and pruning techniques such as pathfinder, minimum spanning tree, and pruning sliced networks were applied.

The R language environment utilizes open-source packages, specifically Bibliometrix and Biblioshiny (28), for conducting bibliometric analysis. Within Rstudio, the dedicated program from the Bibliometrix R package was employed for this purpose. R software along with the R packages “bibliometrix” was employed to analysis highly cited articles. Notably, this tool enables quantitative analysis of bibliographic data (29–31).

## 3 Results

### 3.1 Analysis of annual publication

**Figure 1:**
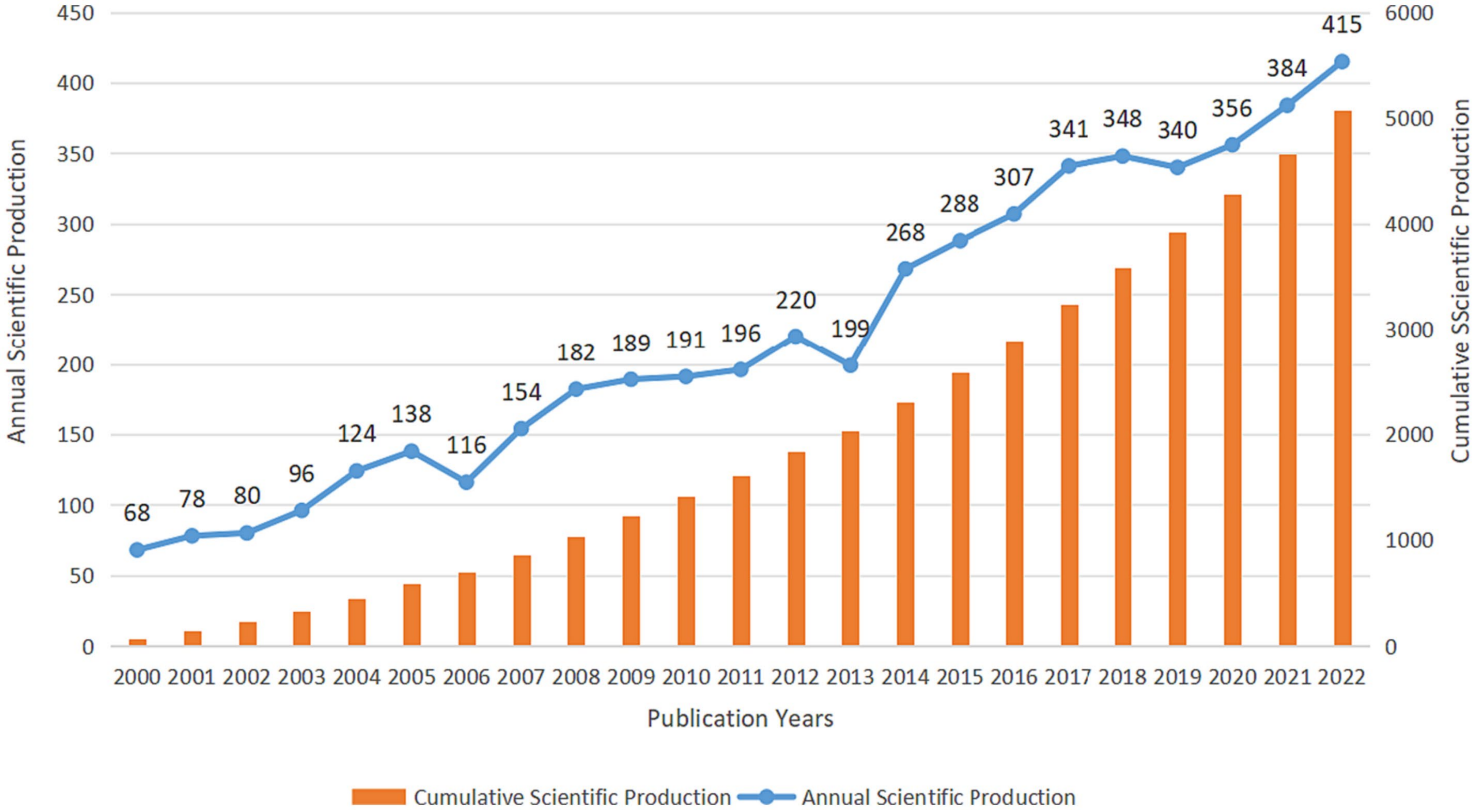
The annual scientific publication of research in the field of cell death after SCI from 2000 to 2022 in the WoSCC database.

5,078 records were retrieved from the WoSCC database from 1 January 2000 to 31 December 2022, including 4,270 (84.09%) articles and 808 reviews (15.91%). Figure 1 shows the annual scientific production of research in the field of cell death after SCI. Initially, limited publications were produced. However, the annual number of publications increased significantly in the past 10 years, reaching a peak in 2022 (*n* = 415).

### 3.2 Analysis of countries/regions

**Figure 2:**
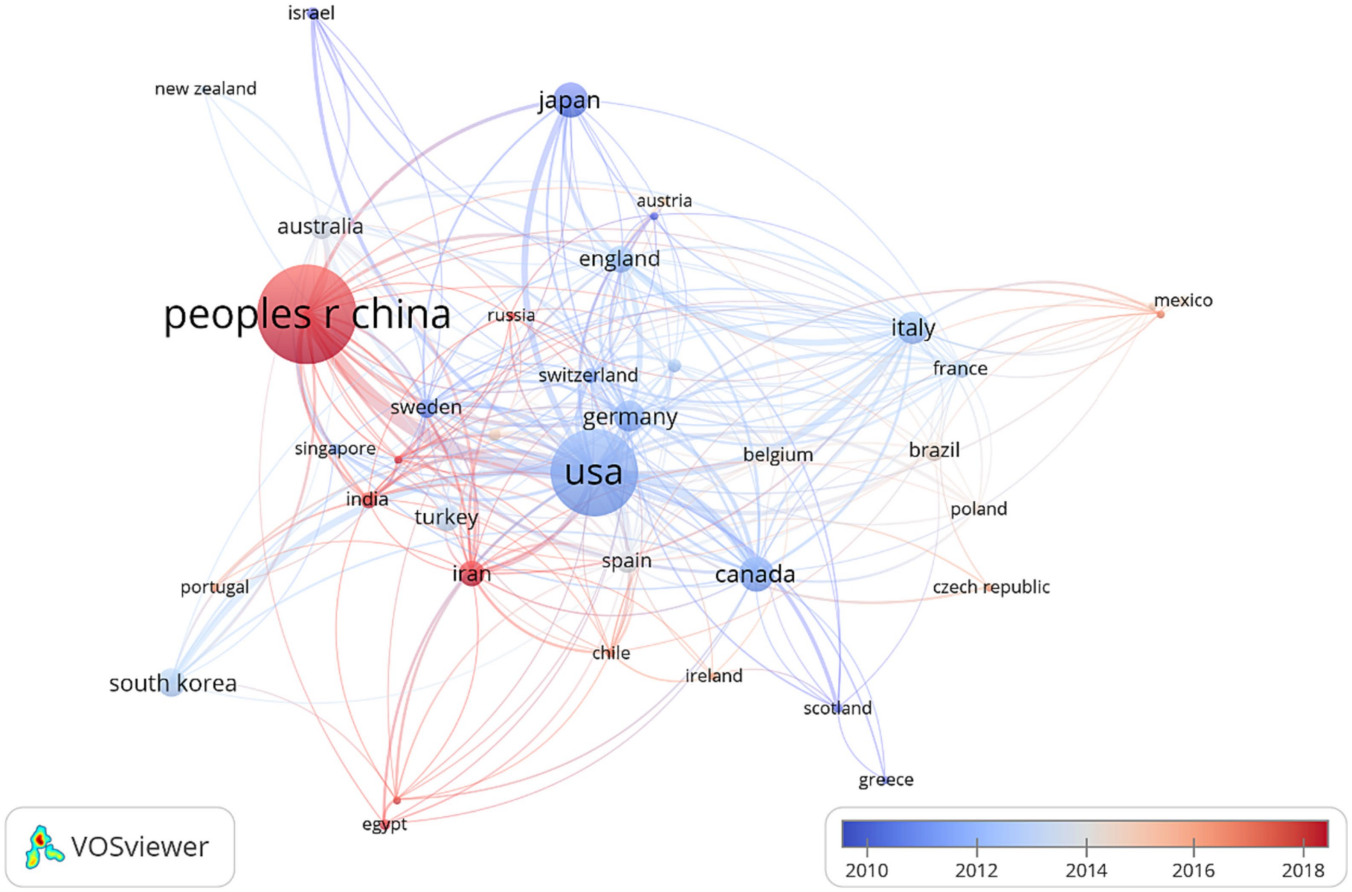
VOSviewer was employed to conduct a country/region co-authorship analysis. The overlay visualization map portrays each node as a country/region, wherein the connections between nodes symbolize the co-authorship associations. The magnitude of each node is directly proportional to the overall count of publications attributed to that particular country. Moreover, the hue of each node corresponds to the average year of appearance, as indicated by the color gradient in the lower right corner of the map.

Analyzing which countries/regions were researching cell death after SCI was crucial. A comprehensive selection encompassing 39 countries/regions, each with a minimum publication count of 10, was incorporated into the analysis. Figure 2 illustrates the central positioning of China (Documents = 1,992) and the United States (Documents = 1,528) within the overlay visualization map. The color gradient displayed in the lower right corner designates countries such as China, India, and Iran with a red hue, while Brazil, Australia, and Spain, among others, are represented by a gray shade.

### 3.3 Analysis of institution

**Figure 3:**
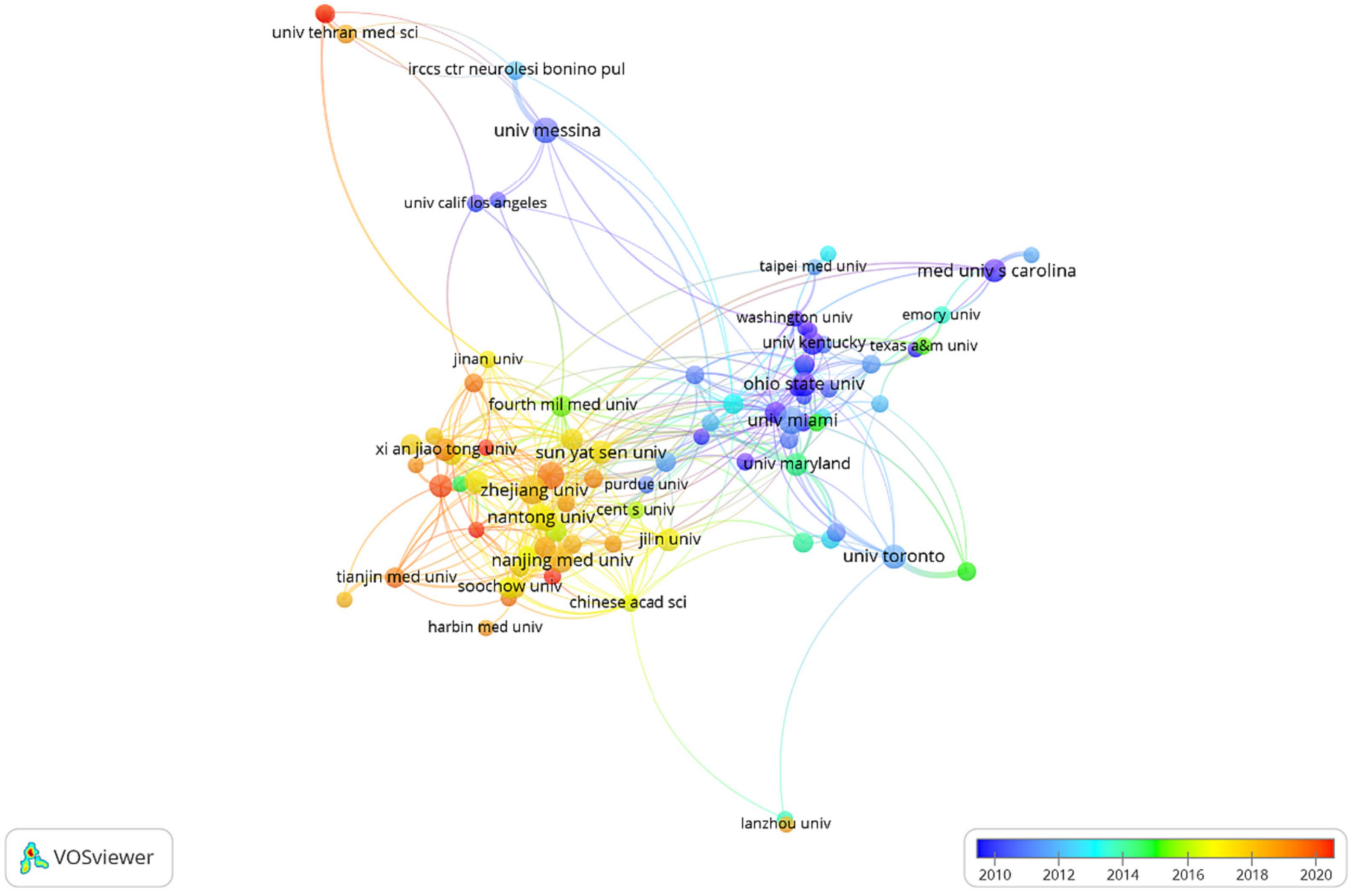
Institution co-authorship analysis by VOSviewer. Each organization is represented as a node, and the node size is proportional to the sum of publications.

The network map of the institution’s co-authorship analysis is shown in Figure 3. A total of 87 institutions, each having a minimum of 20 publications, were considered for inclusion in the study. Regarding the number of documents, Wenzhou Medical University contributed the most papers (Documents = 114), followed by the University of Messina (Documents = 88), and Nanjing Medical University (Documents = 85). It becomes apparent that the level of international collaboration between institutions from diverse countries was not sufficiently extensive, with the majority of collaborations occurring among domestic institutions.

### 3.4 Analysis of author

**Figure 4:**
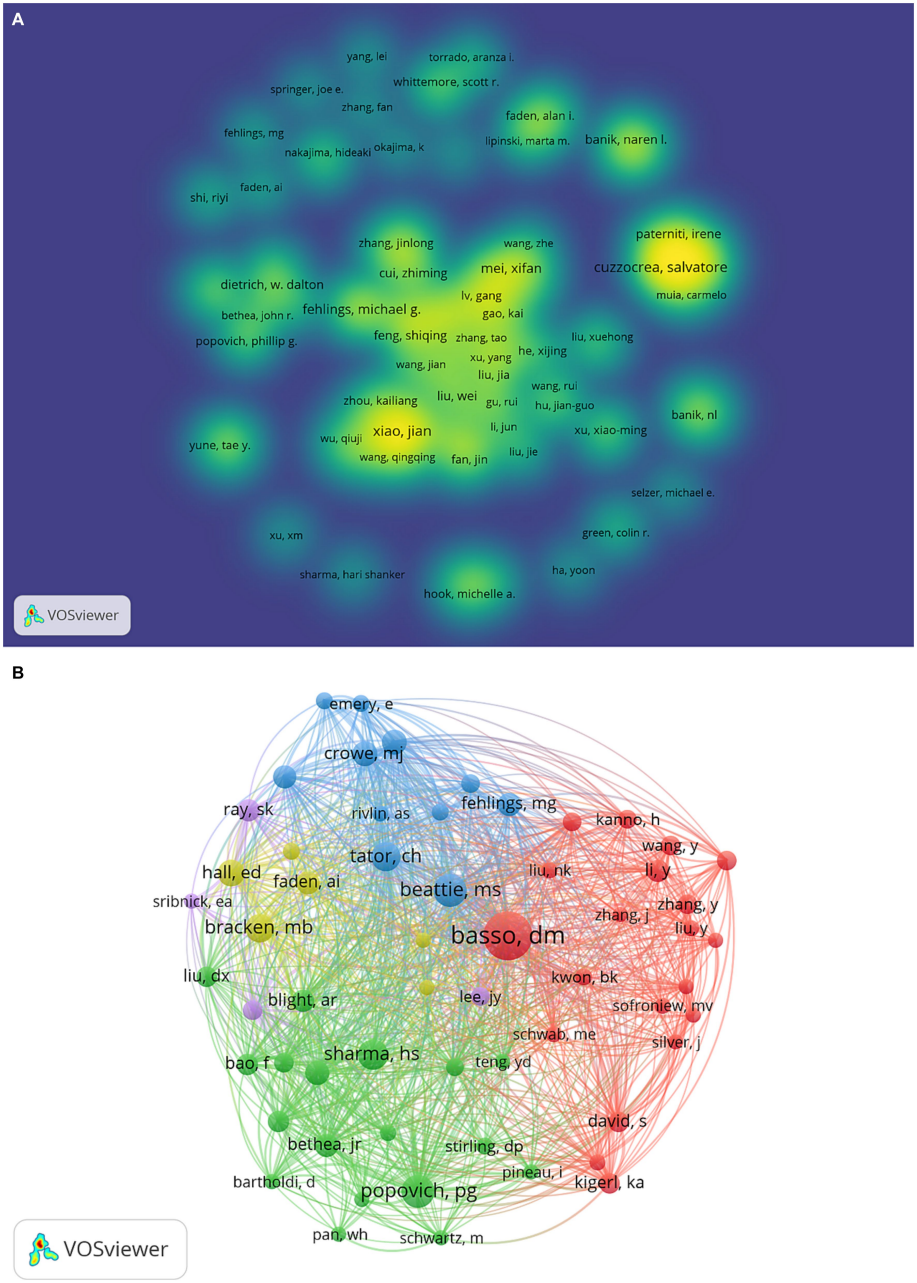
**(A)** The VOSviewer software was utilized to analyze author co-authorship. The resulting cluster density map visually represents authors who share a close relationship, as they are grouped within clusters of the same color. **(B)** The VOSviewer software was employed to perform an analysis of author co-citation. The resulting network visualization map depicts each author as a node, while the lines connecting these nodes represent the co-citation relationships between them.

A cluster density map illustrating the examination of author co-authorship was produced utilizing VOSviewer (Figure 4A). Among the 161 authors (more than 10 papers) included in the analysis, Salvatore Cuzzocrea from the University of Messina had the highest number of publications, followed by Esposito Emanuela from the University of Messina, and Xiao Jian from the University of Chinese Academy of Sciences. Furthermore, authors with a strong collaborative relationship were grouped in clusters of the same color, resulting in a total of 11 author clusters. Notably, the majority of these clusters were comprised of authors from China. In the context of author co-citation analysis, a total of 59 authors meeting the criterion of at least 200 citations were encompassed (Figure 4B). Notably, the authors Basso DM, Beattie MS, and Popvich PG emerged as the top three contributors, exhibiting the total link strength.

### 3.5 Journal publications and citation analysis

**Figure 5:**
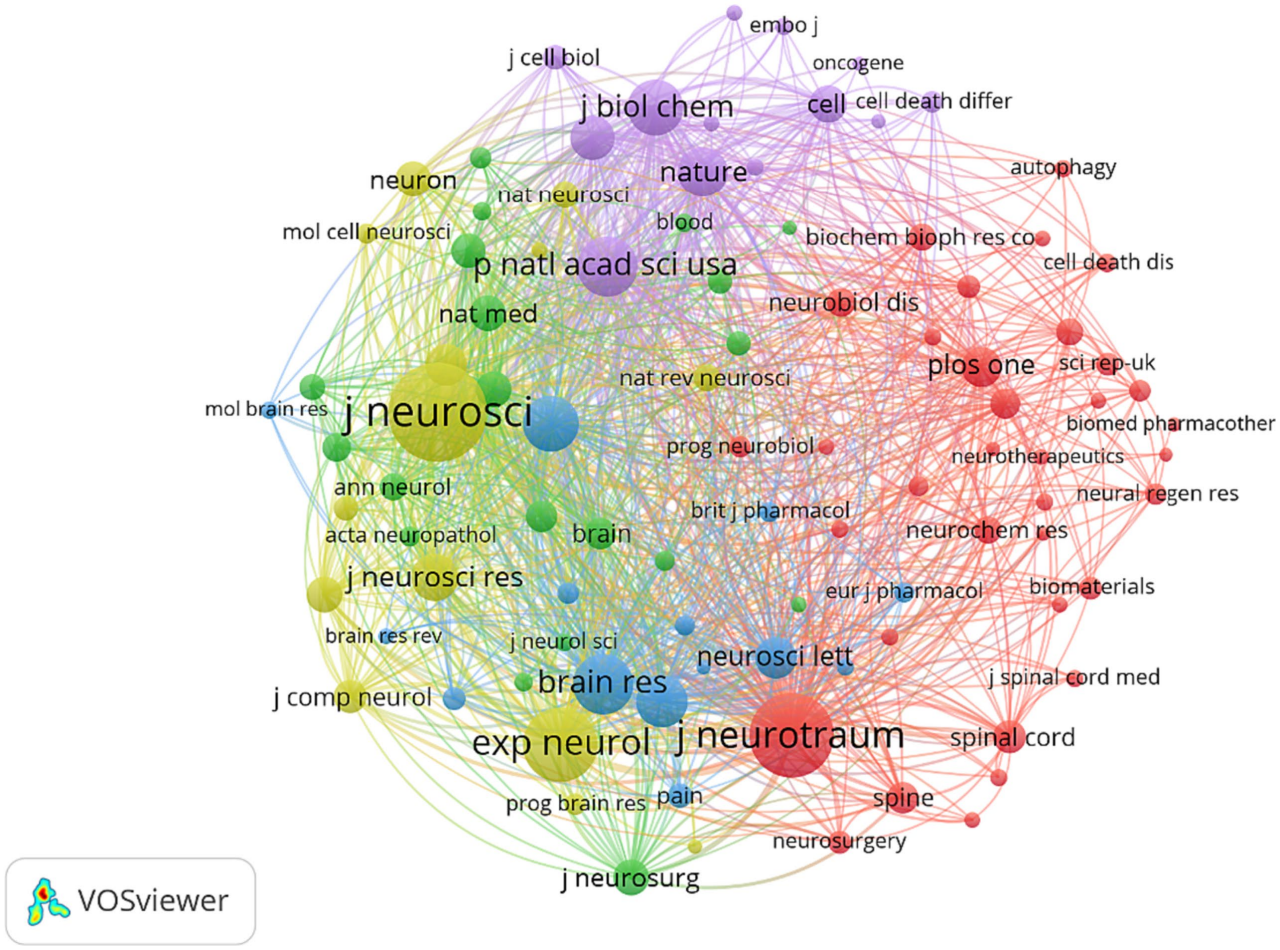
Journal co-citation analysis by VOSviewer. Each node represents a journal and the node size is proportional to the number of co-citations by that journal.

In terms of documents, the “Journal of Neurotrauma” was the most prominent (Documents = 234), followed by “Neural Regeneration Research” (Documents = 131), and “Experimental Neurology” (Documents = 114), as listed in Table 1. As shown in Figure 5, a co-citation map of journals was generated using VOSviewer, with a minimum citation threshold of 600. Out of the total, 96 journals successfully met this criterion. Notably, the “Journal of Neuroscience” emerged as the most frequently co-cited journal, with a total link strength of 13106.21, followed by the Journal of Neurotrauma, and Experimental Neurology.

**Table 1:** Top 10 journals of publications.

Ranking	Journal	Documents	% of 5,078	IF 2022	JCR quartile 2022
1	Journal of Neurotrauma	234	4.61	4.2	Q2
2	Neural Regeneration Research	131	2.58	6.1	Q1/Q2
3	Experimental Neurology	114	2.24	5.3	Q1
4	Brain Research	102	2.01	2.9	Q3
5	Neuroscience	89	1.75	3.3	Q3
6	Neuroscience Letters	81	1.59	2.5	Q3
7	Molecular Neurobiology	79	1.56	5.1	Q1/Q2
8	Neurochemical Research	78	1.54	4.4	Q2
9	Journal of Neurochemistry	73	1.44	4.7	Q2
10	Journal of Neuroinflammation	72	1.42	9.3	Q1

### 3.6 Highly cited papers

Highly cited publications can help researchers identify relevant research topics that are relevant to them through the content of highly cited publications. Table 2 shows the top 10 highly cited papers in the WoSCC database based on total citation count. The most cited paper (Total citations = 1,598) was “Identification of two distinct macrophage subsets with divergent effects causing either neurotoxicity or regeneration in the injured mouse spinal cord,” by Kigerl KA from the Ohio State University in the USA, followed by Pacher P from the National Institutes of Health (Total citations = 1,521) and Mattson MP from the National Institute on Aging (Total citations = 1,230). We concluded that these top papers mainly focus on the mode of cell death, mechanism research, and functional recovery. In addition, we selected the top 10 highly cited papers based on the total citations per year, as shown in Table 3. We concluded that the focus of these papers was similarly to the most globally cited documents by the total citations.

**Table 2:** The most globally cited documents by the total citations.

Ranking	Title	Journal	First author	Publish year	Total citation
1	Identification of two distinct macrophage subsets with divergent effects causing either neurotoxicity or regeneration in the injured mouse spinal cord	J Neurosci	Kigerl KA	2009	1,598
2	The endocannabinoid system is an emerging target of pharmacotherapy	Pharmacol Rev	Pacher P	2006	1,521
3	Apoptosis in neurodegenerative disorders	Nat Rev Mol Cell Bio	Mattson MP	2000	1,230
4	Epidemiology, demographics, and pathophysiology of acute spinal cord injury	Spine	Sekhon LHS	2001	918
5	Traumatic spinal cord injury	Nat Rev Dis Primers	Ahuja CS	2017	906
6	Role of the immune system in chronic pain	Nat Rev Neurosci	Marchand F	2005	858
7	Inflammation and its role in neuroprotection, axonal regeneration and functional recovery after spinal cord injury	Exp Neurol	Donnelly DJ	2008	717
8	Neuroinflammation: the devil is in the details	J Neurochem	Disabato DJ	2016	710
9	Microglia activated by IL-4 or IFN-gamma differentially induce neurogenesis and oligodendrogenesis from adult stem/progenitor cells	Mol Cell Neurosci	Butovsky O	2006	689
10	Central nervous system injury-induced immune deficiency syndrome	Nat Rev Neurosci	Meisel C	2005	680

**Table 3:** The most globally cited documents by the total citations (TC) per year.

Ranking	Title	Journal	First author	Publish year	Total citation per year
1	Traumatic spinal cord injury	Nat Rev Dis Primers	Ahuja CS	2017	129.43
2	Traumatic Spinal Cord Injury: an Overview of Pathophysiology, Models and Acute Injury Mechanisms	Front Neurol	Alizadeh A	2019	106.6
3	Identification of two distinct macrophage subsets with divergent effects causing either neurotoxicity or regeneration in the injured mouse spinal cord	J Neurosci	Kigerl KA	2009	106.53
4	Neuroinflammation and Central Sensitization in Chronic and Widespread Pain	Anesthesiology	Ji RR	2018	99.5
5	Neuroinflammation: the devil is in the details	J Neurochem	Disabato DJ	2016	88.75
6	The endocannabinoid system as an emerging target of pharmacotherapy	Pharmacol Rev	Pacher P	2006	84.5
7	The Biology of Regeneration Failure and Success After Spinal Cord Injury	Physiol Rev	Tran AP	2018	75.67
8	Muscle wasting in disease: molecular mechanisms and promising therapies	Nat Rev Drug Discov	Cohen S	2015	74.78
9	The far-reaching scope of neuroinflammation after traumatic brain injury	Nat Rev Neurol	Simon DW	2017	72.71
10	Astrocytes: Key Regulators of Neuroinflammation	Trends Immunol	Colombo E	2016	66.38

### 3.7 Keywords analysis

#### 3.7.1 Co-occurrence analysis of keywords

**Figure 6:**
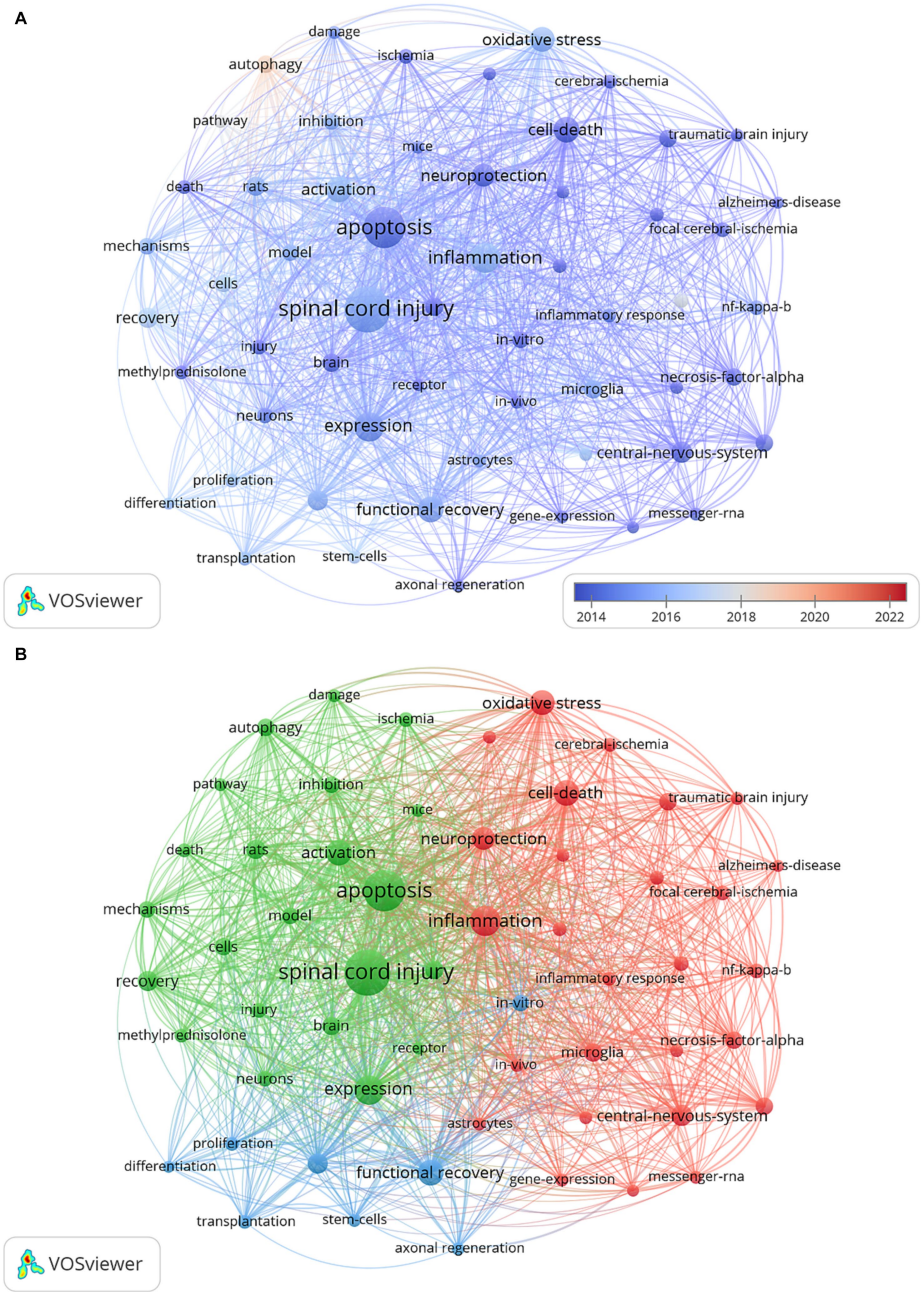
Co-occurrence analysis of keywords: **(A)** The visualization map of keyword co-occurrence analysis includes an overlay, where the color of each node corresponds to the average annual yield, as indicated by the color gradient in the lower right corner. Nodes marked with purple or blue represent keywords that appeared earlier, while those coded in red represent current research focuses. **(B)** The network visualization map of keyword co-occurrence analysis demonstrates the clustering of closely related keywords, assigned the same color within each cluster.

The keywords in literature serve to identify specific data items, briefly and accurately describe the article topic and are primarily used for indexing or cataloging (32). Therefore, analyzing keyword changes can reveal the characteristics and evolution trends of publications. The minimum amounts of occurrences of a keyword were set as 150. There were 57 keywords that met the threshold. The most frequently occurrences keyword was “spinal cord injury” (Occurrences = 2,123). Finally, we constructed a keyword analysis merging a coexistence network using all. The keyword overlay map utilized color changes in keyword nodes to represent the evolution of keywords over time. According to Figure 6A, “autophagy,” was new keywords that had emerged in recent years. In addition, the center of the keyword density map represented the set of keywords with the highest frequency of occurrence. Figure 6B illustrated that the keyword “spinal cord injury” had the highest frequency, followed by “apoptosis,” “inflammation,” “activation,” “expression,” “oxidative stress,” “cell death,” and “function recovery.”

#### 3.7.2 Cluster analysis of keywords

**Figure 7:**
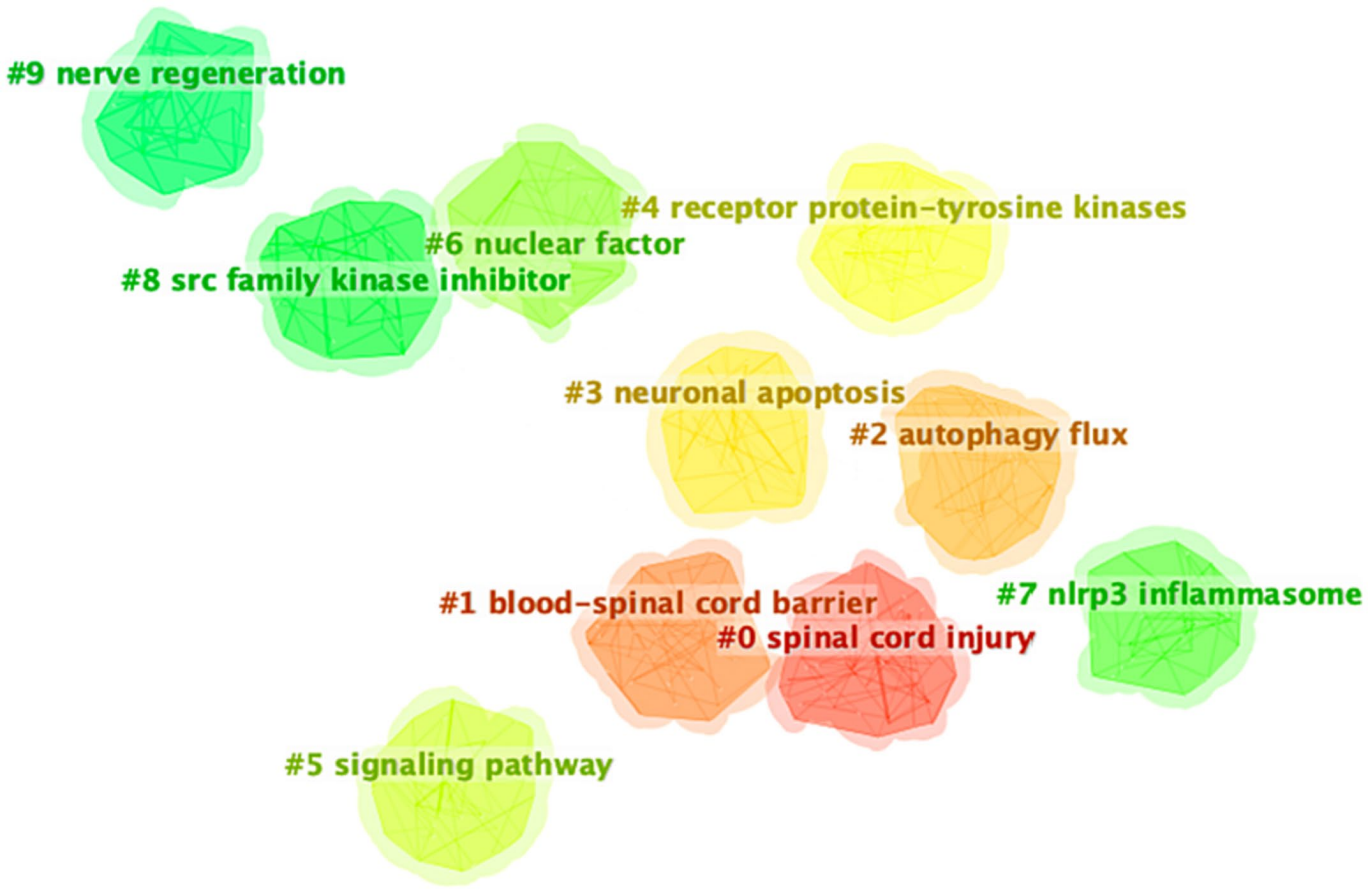
Keyword cluster map.

The retrieved keywords were grouped into 10 clusters based on their similarity, providing a partial representation of the knowledge structure in this topic. As showcased in Figure 7, 10 major clusters formed after running the software. The first cluster (#0), labeled as “spinal cord injury,” focused on apoptosis, expression, activation, recovery, and model. Cluster (#1), labeled as “blood-spinal cord barrier,” focused on inflammatory response; axon regeneration; promotes; progenitor cells; migration. Cluster (#2), labeled as “autophagy flux,” focused on functional recovery; pathophysiology; microglial activation; involvement; neuroinflammation; olfactory ensheathing cells. Cluster (#3), labeled as “neuronal apoptosis,” focused on tnf-alpha; lipid-peroxidation; reactive oxygen species; gene; cytochrome-c. Cluster (#4), labeled as “receptor protein-tyrosine kinases,” focused on cell death; rats; protein; stem-cells; macrophages. Cluster (#5), labeled as “signaling pathway,” focused on regeneration; neurons; differentiation; survival; degeneration. Cluster (#6), labeled as “nuclear factor,” focused on apoptosis; inhibition; damage; growth; protects. Cluster (#7), labeled as “nlrp3 inflammasome,” focused on oxidative stress; neuropathic pain; nitric oxide synthase; graded model; peroxynitrite. Cluster (#8), labeled as “src family kinase inhibitor,” focused on *in-vivo*; neurotrophic factor; gene-expression; delivery; secondary damage. Cluster (#9), labeled as “nerve regeneration,” focused on pathway; autophagy; up-regulation; identification; rapamycin.

#### 3.7.3 Keywords with the strongest citation bursts

**Figure 8:**
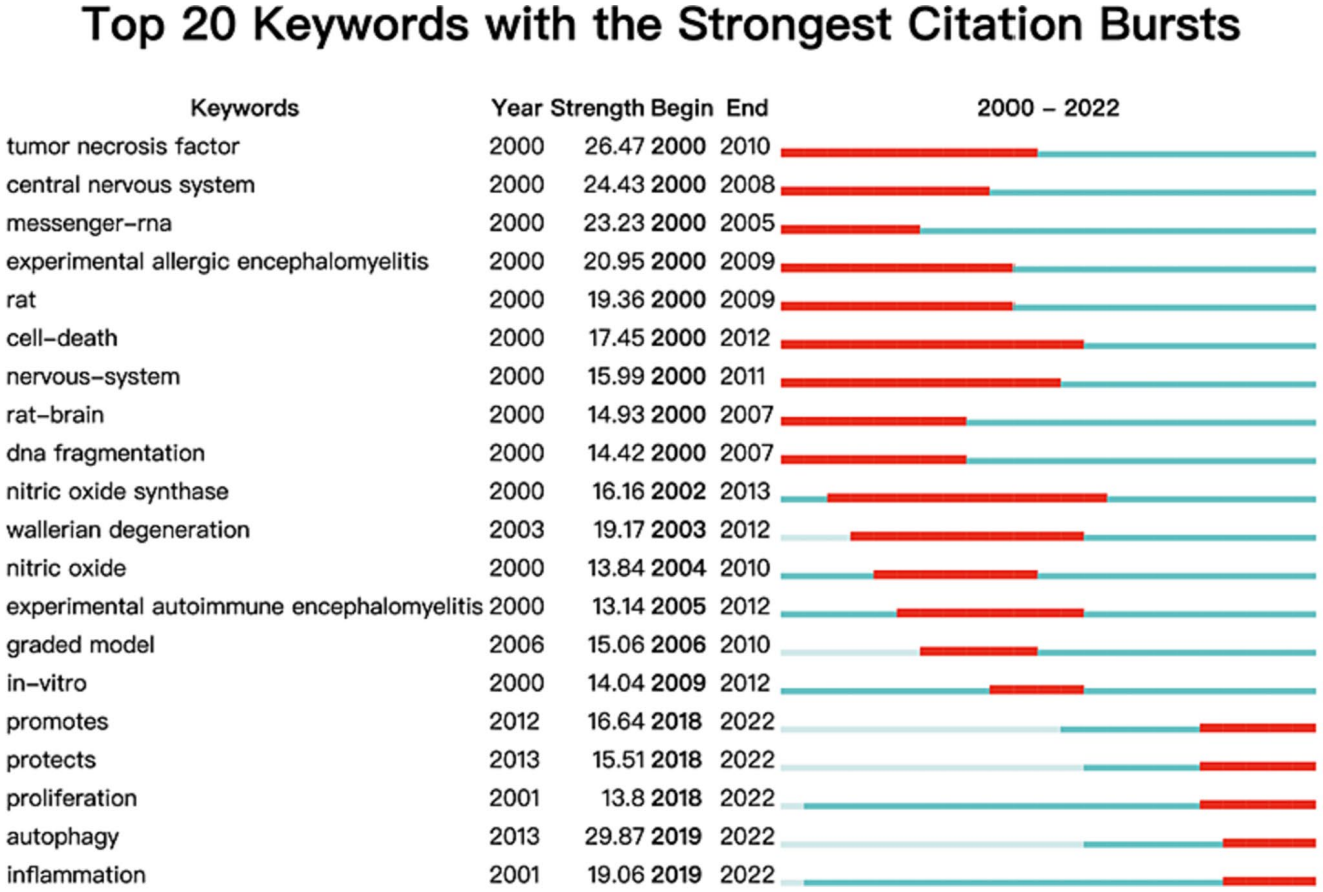
Keywords with strongest citation bursts by CiteSpace. In the first column, “keywords” represented the theme terms. In the second column, “year” represented the year in which a theme term first appeared. In the third column, “strength” represented the citation bursts strength of these terms. In the fourth column, “begin” represented the starting year of a theme term burst. In the fifth column, “end” represented the end year of a theme term burst.

CiteSpace’s burst detection feature, which identifies significant keyword changes over a certain period (33), was used to screen the top 20 keywords. The impact of keywords amplified according to the rise in the “strength” value. As shown in Figure 8, the keywords that had the highest strength were “autophagy” (29.87), followed by “tumor necrosis factor” (26.47), and “central nervous system” (24.43). Besides, the bright blue region denoted the duration of the study, while the red areas indicated the start and end of a burst. Notably, the longest-bursting keyword was “cell death,” which began in 2000 and ended in 2012. Keywords such as “promotes,” “protects,” “proliferation,” “autophagy,” and “inflammation” continued to burst until 2022, representing research hotspots in the last 5 years.

### 3.8 Literature co-citation analysis

**Figure 9:**
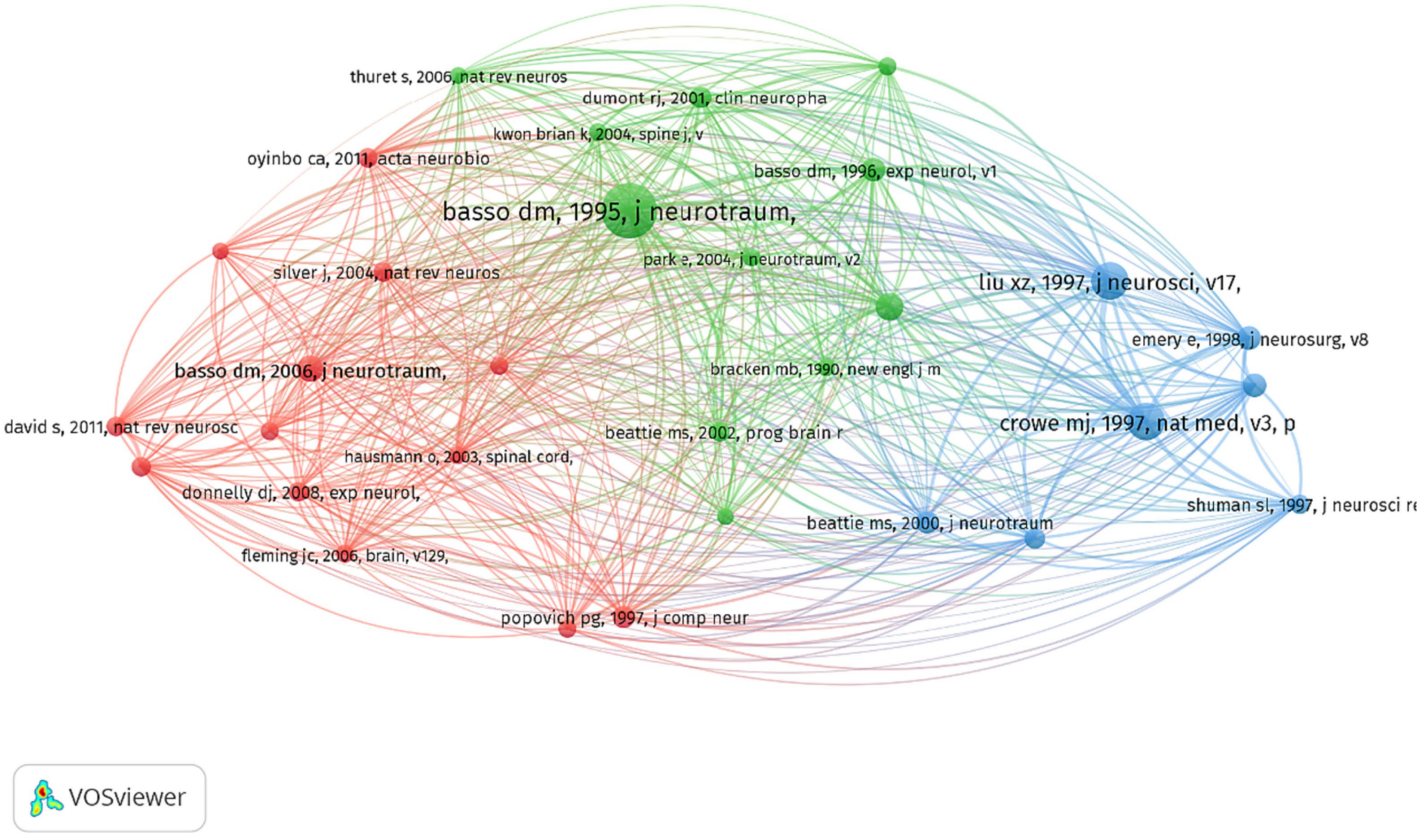
Literature co-citation analysis. Clusters are represented by color, nodes by size, and co-citations by link thickness.

To further examine literature pivotal in promoting research in the field of cell death after SCI, we also used VOSviewer to conduct a co-citation network analysis of cited references. Finally, 31 references with more than 150 citation frequencies were selected as the co-citation network nodes, forming 3 clusters, as shown in Figure 9. Overall, the articles in the green cluster focus on valid locomotor rating scales for rat. Basso (34) proposed that the BBB locomotor rating scale for testing SCI-related behavioral consequences is widely used in SCI rats. In addition, the blue cluster focuses on articles related to apoptosis after SCI. Crowe and Liu (2, 35) were at the core of the co-citation network in the blue cluster. Research in the green cluster mainly focused on the valid locomotor rating scale for SCI mice. Of those, Basso (36) was the core of the co-citation network in the red cluster.

## 4 Discussion

SCI is a traumatic injury that threatens human health. Even though SCI always leads to cell death, many challenges persist, necessitating further investigation into research on cell death following SCI. Insights into these questions can be gained from a variety of bibliometric analyses. The bibliometric analysis of literature practically captures characteristics and hotspots, thereby facilitating rapid comprehension of unfamiliar materials.

In general, annual scientific production counts serve as a reliable measure to gauge the research focus of scholars within a specific discipline. A careful examination of the yearly publication figures reveals a notable upward trajectory spanning the last 23 years. This trend can be roughly categorized into two distinct periods: a period of gradual growth from 2000 to 2011, followed by a period of rapid expansion from 2012 to 2022. Prior to 2012, the annual publication count exhibited a relatively sluggish increase, with the number of papers per year remaining below 200.

The analysis of the top 10 countries/regions with the highest productivity in the study of cell death after SCI, as presented in Figure 2, reveals a concentration of these entities in the Northern Hemisphere continents, particularly in East Asia, North America, and various European countries. Statistical data indicates that China holds the foremost position in terms of the overall publication count in the field of SCI-related cell death research, closely followed by the United States. Undoubtedly, China and the United States have made the most substantial contributions in this area. The underlying factors for this prominence can be attributed to substantial governmental investments (37).

The top three institutions that have published the highest number of documents related to cell death after SCI are Wenzhou Medical University, University of Messina, and Nanjing Medical University. This observation is supported by the data presented in Figure 3, which indicates that China possesses the most productive institutions in this domain. This finding may partially account for China’s consistently high publication output. Furthermore, the analysis of countries reveals that a majority of Chinese institutions exhibit a larger average annual yield value, indicating their recent entry into this field. Furthermore, it is noteworthy to mention that the level of international collaboration among institutions from diverse countries was insufficient, with the majority of collaborations occurring primarily within domestic institutions.

The inclusion of authors’ analyses is an essential aspect of bibliometric analysis, taking into account various factors. Notably, Salvatore Cuzzocrea and Esposito Emanuela from the University of Messina have made significant contributions by producing the most extensive body of research on cell death following SCI. Specifically, Salvatore Cuzzocrea’s research primarily centers on investigating the mechanisms of apoptosis subsequent to injury (38, 39). In addition, she also focused on necroptosis (40) and autophagy (41) of cell death to SCI. Esposito Emanuela is mainly focused on mechanisms of autophagy, and inflammation after SCI (41, 42). In conclusion, these scholars were regarded as having distinct and essential contributions in the domain of cell death following spinal cord injury, whether in terms of quantity or quality.

Journal analysis is a prevalent component of bibliometric analysis, offering valuable insights to researchers in selecting appropriate journals for their study submissions. Previous bibliometric studies have consistently recognized the significance of journal analysis (22, 23, 43), thus warranting an examination of the attributes exhibited by the most widely recognized journals within this domain. As can be seen from Table 1, the “Journal of Neurotrauma,” “Neural Regeneration Research,” and “Experimental Neurology” were the top three most popular journals involved in the publication of research on cell death after SCI. Therefore, it can be reasonably inferred that forthcoming advancements concerning cell death subsequent to SCI are more inclined to be disseminated through these scholarly journals. Furthermore, with regard to the categorization of the top 10 journals, a majority of them pertain to clinical neurology, critical care medicine, neurosciences, and cell biology, thereby mirroring the primary research orientations and emphases within this field. In addition, a journal co-citation analysis was performed to ascertain the principal journals in the field. The analysis revealed that the “Journal of Neuroscience,” “Journal of Neurotrauma,” and “Experimental Neurology” were the top three journals with the highest total link strength. This outcome can be attributed primarily to the publication of studies of exceptional quality within these journals. Consequently, it is plausible that forthcoming advancements pertaining to cell death after SCI may also be found within the pages of these journals.

Keyword analysis is a fundamental aspect of academic papers as it serves to depict the prevailing research interests, facilitate comprehension of the evolutionary trajectory, and enable research prediction. In this section, we have employed two bibliometric software tools to conduct a thorough analysis. Firstly, VOSviewer has been utilized to generate a keyword co-occurrence network map, wherein keywords exhibiting strong associations are grouped together and assigned the same color. Figure 6B illustrates the classification of these keywords into three distinct categories. We could find that current research directions for cell death after SCI mainly focus on studies on the mode of cell death (10, 44), mechanism research (45, 46), and functional recovery (19, 47). In addition, the overlay visualization map of keywords co-occurrence analysis in VOSviewer facilitates the assignment of distinct colors to keywords based on their average annual yield. Nodes labeled with purple hues indicate keywords that have been observed relatively earlier, while keywords represented by red hues signify current research focuses. As depicted in Figure 6A, during the initial phase, the investigation of cell death primarily centered around the concept of “apoptosis.” Conversely, keywords associated with autophagy exhibited a comparatively higher average annual yield, suggesting a shift in the prevailing research focus toward autophagy within the field. In relation to keywords exhibiting the most prominent citation bursts, it likewise exhibited analogous shifts in terms of keywords. The temporal analysis of the keyword displaying the most pronounced burst indicates that starting from the late 2000s, programmed cell death in SCI garnered significant attention, primarily owing to apoptosis being regarded as the sole form of programmed cell death. Presently, autophagy has emerged as the prevailing area of research interest. Furthermore, as shown in Figure 7, the cluster map conducted by CiteSpace also mainly focus on the mode of cell death (e.g., neuronal apoptosis, autophagy), the regulatory mechanism (blood-spinal cord barrier, signal pathway, nlrp3 inflammasome, etc.), and nerve regeneration. In the context of literature co-citation analysis, prior research has primarily concentrated on investigating the regulation of apoptosis following SCI (2, 35). Nevertheless, the existence of reciprocal referencing within the scientific literature indicates that apoptosis is not an isolated phenomenon, but rather a complex network of interconnected associations that continually expands. Consequently, future studies will continue to emerge based on the findings of previous research.

In addition, the ultimate phase of a cell’s lifespan is referred to as cell death, which can be triggered by either exogenous or endogenous cytotoxicity. Initially, cell death was classified into necrosis and apoptosis. Necrosis entails the passive and inadvertent death of cells due to environmental disturbances and the discharge of inflammatory agents. In contrast, apoptosis is regarded as an active and orchestrated mechanism of cellular self-deconstruction that does not involve the release of cytoplasmic contents into the extracellular milieu. Apoptosis was observed in various cell types, including neurons, astrocytes, oligodendroglia, and microglia, within the rat spinal cord following injury (48). Notably, a predominant presence of apoptotic oligodendrocytes was identified within the white matter longitudinal tracts (49). The study reported the occurrence of apoptotic cells in the early post-injury phase, particularly within the white matter region of the spinal cord. Neuronal apoptosis commenced at the onset of 4 h post-injury and reached its peak 8 h thereafter. Conversely, glial apoptosis initiated at the same 4-h mark and culminated 24 h post-injury. However, oligodendrocytes only initiated apoptosis at the 24-h mark following injury, attaining its pinnacle 8 days later (35, 50). Microglia, on the other hand, exhibited relatively low levels of apoptosis during the initial 24 h and first 5 days post-injury, with a gradual increase observed thereafter, peaking at the eighth day (50). Moreover, the presence of apoptotic cells predominantly localized in the periphery of the viable tissue surrounding the central region of the injured spinal cord provides a potential explanation for the progressive enlargement of the lesion area (51).

Furthermore, recent advancements in scientific research have unveiled diverse modalities of programmed cell death, encompassing autophagy, necroptosis, pyroptosis, and ferroptosis. Autophagy serves as a mechanism for mitigating intracellular and extracellular stress. Prior research has demonstrated the inhibition of autophagy following SCI, resulting in neuronal demise and deterioration (3, 52). However, the precise impact of autophagy on various stages of SCI remains uncertain (10); Necroptosis, a regulated form of necrosis mediated by receptor-interacting protein kinases 1 and 3 (RIPK1 and RIPK3) (53, 54), is triggered after SCI and is believed to play a role in the demise of neuronal and glial cells (55, 56). Furthermore, mounting evidence suggests a connection between necroptosis and inflammation in the context of SCI (57). The regulation of pyroptosis is mediated through the activation of caspase-1, caspase-11, caspase-4, and caspase-5 signaling pathways, resulting in a cascade of inflammatory reactions. Consequently, the inhibition of pyroptosis-induced cell death, along with the modulation of the inflammasome component of the inflammatory response, has demonstrated potential as a therapeutic strategy for the treatment of SCI in forthcoming clinical interventions (58). In recent years, a growing number of studies have focused on ferroptosis’ role in SCI. Ferroptosis, a recently identified form of programmed cell death that does not involve tissue necrosis, exhibits a significant association with excitotoxicity-induced neuronal demise. It is widely acknowledged that the acute phase of SCI leads to the destruction and degradation of numerous blood cells and hemoglobin, consequently causing bleeding, hemolysis, and a substantial elevation in iron ion concentrations within the affected region (59, 60). The neuronal impairment caused by the intracellular buildup of unbound iron can lead to iron metabolism disorders (61). Reactive oxygen species are generated through the Fenton reaction, wherein H_2_O_2_ reacts with polyunsaturated fatty acids in the lipid membrane, resulting in the depletion of reduced glutathione, inactivation of GXP4, damage to the cell membrane, and ultimately triggering ferroptosis (62). The relationship between ferroptosis and secondary SCI, along with inflammation, immunity, and chronic injury, exhibits a significant correlation (63). Consequently, ferroptosis has been acknowledged as a pivotal therapeutic target in the treatment of spinal cord injuries (45, 64). The notion has been put forth that paraptosis, a form of programmed cell death, is characterized by mitochondrial dilation and/or endoplasmic reticulum swelling resulting from cytoplasmic vacuolation. Unlike apoptosis, paraptosis does not depend on caspases or exhibit apoptotic morphologies (65). Nevertheless, the understanding of paraptosis in the context of SCI remains limited, considering it is a recently identified form of programmed cell death.

## 5 Limitation

This study has certain limitations. First, the number of databases limited this study’s scope. For example, data from PubMed, Embase, and Cochrane Library, which are other pertinent search engines, were disregarded. In addition, the inclusion criteria for this study were limited to publications in the English language, thereby potentially excluding relevant studies conducted in other languages. Nevertheless, it is widely acknowledged that WoSCC is the most appropriate and recommended database for conducting bibliometric analysis, as it encompasses a comprehensive range of information within a specific field. Second, in order to conduct a comprehensive analysis of the complete annual data, the scope of included publications was restricted to the period between 2000 and 2022, with no consideration given to publications released in 2023. Consequently, potential hotspots within the cell death after the SCI field in 2023 may have been overlooked. Nevertheless, by examining publications spanning a 23-year panorama, the overall publication trends and emerging research hotspots in cell death after SCI were identified. Lastly, obtaining all the relevant literature in this field is challenging due to the wide range of keywords. We posit that incorporating logical operators into the search strategy has the potential to significantly enhance the precision of the retrieved outcomes.

## 6 Conclusion

This bibliometric study has unveiled the comprehensive publication patterns and dynamic research trends on a global scale spanning two decades. Additionally, it has identified potential collaborators, hotspots, and forthcoming research directions in the field, thereby providing valuable guidance for future investigations in the field of cell death after SCI. Furthermore, the findings of this study will serve as a valuable resource for clinicians, researchers, industry collaborators, policymakers, and other stakeholders.

## Data availability statement

The original contributions presented in the study are included in the article/supplementary material, further inquiries can be directed to the corresponding authors.

## Author contributions

KH: Funding acquisition, Software, Writing – original draft. HY: Investigation, Software, Writing – original draft. JZ: Methodology, Writing – review & editing. LW: Methodology, Supervision, Writing – review & editing. DH: Validation, Writing – review & editing, Funding acquisition. RM: Funding acquisition, Supervision, Writing – review & editing.
